# Dr. McLaughlin’s Microbic Theory of Dengue

**Published:** 1886-12

**Authors:** R. Menger

**Affiliations:** San Antonio, Texas


### DR. McLAUGHLIN'S MICROBIC THEORY OF DENGUE FEVER.

READ BEFORE THE WEST TEXAS MEDICAL ASSOCIATION.

*Reported by R. Menger, M. D., San Antonio, Texas.*

For Daniel's Texas Medical Journal.

NOW, that dengue fever is again the topic of the day, allow me to present to you the late discoveries made by Dr. J. W. McLaughlin, concerning the microbian theory of dengue.

Dr. McLaughlin made, during a six months uninterrupted work, very interesting and elaborate investigations with blood taken directly from dengue fever patients and submitted, under all possible precautions against external contamination, to a rigid microscopical examination, using the best instruments and the most approved methods of staining, etc. The Doctor found, invariably, myriads of smallest, round, or oval-shaped bodies, not alone in the blood-serum, but especially in the blood corpuscles, often counting 10, 15, 20 and more in one blood cell. This is a discovery heretofore not made known; and, that they are organisms of the bacteria type, is approved, not alone by Koch, in Berlin, to whom the Doctor sent microscopic slides, but also by one of the most distinguished pathologists and microscopists in the south, Dr. H. D. Schmidt, pathologist to the Charity Hospital at New Orleans.

We are all more or less acquainted with the bacteriæ of other diseases; in fact, there are but few diseases in which organisms have not been discovered. We know that micrococci are found in ordinary pus, in abscesses of pyæmia, in old ulcerative processes, in old, simple and complicated wounds, in erysipelas, measles, scarlatina, cerebro-spinal meningitis, dysentery, yellow fever, diphtheria, hydrophobia, in swine plague, etc., and that other organisms called bacilli are also found in various diseases, and have been accordingly classified, for instance: the bacillus subtilis, the bacillus of human saliva, the bacillus anthracis, or microbe of malignant pustule, baccilus tuberculosis, bacillus typhosus of typhoid fever, bacillus lepræ of leprosy, bacillus malariæ, bacilli of syphilis, glanders, pertussis, septicæmia and cholera. Then we have a "Spirillum," discovered in blood of patients suffering with relapsing fever, but only during the febrile paroxysms, and disappearing in the intermission and through convalescence.

It is also well known that we find myriads of bacteriæ, either micrococci, bacilli or other types of organisms outside of the human system, in liquid and solid substance, especially such undergoing decomposition, and also in the air. Now, is it not very possible for such organisms to enter the human system, multiply there under certain favorable conditions of the system to enormous quantities and produce various abnormal functions of different organs? May this be the case in dengue fever or not, the fact remains that bacteria exist in dengue blood in large quantities and that by their presence the blood contains "foreign bodies,"—so to say, certainly bodies *which do not belong there!* As example, I may relate to those absolutely doubting the microbien theory in disease, a case of trichinosis. What are trichinæ? They are such small organisms (although by far not as small as the largest type of bacteriæ) that it takes a good microscope to detect them. Now, these minute organisms, the trichinæ, also act somewhat like a foreign body in the system, infecting and affecting especially the muscular system. Whoever saw a genuine case of trichinosis knows only too well the excruciating pain and other complications the patient has to suffer from these diminutive micro-organisms!

For my part, I would not lay much stress on *all* of the discoveries made of micro-organisms. I would only believe such diseases to have been caused by them, as those where the investigations had been made with the utmost care and the investigator known to be a thoroughly competent microscopist.

I have not the time to go into a particular detail concerning the microbien theory in general, but, as I had been corresponding with Dr. McL. during his entire investigations, and to prove my assertions, I may be allowed to recite to you several paragraphs of Dr. McLaughlin's pamphlet, entitled, "Researches into the Ætiology of Dengue Fever."

This is a most interesting report, containing many entirely new observations, but I refer you to the original for its entire perusal. I will show you, at the close of this paper, two micro-photos of dengue blood, made recently, from slides of Dr. McLaughlin.

On page 3 Dr. McLaughlin describes the mode of obtaining the blood as follows:

1st. "Blood, which was obtained from living subjects during the various stages of dengue, was microscopically examined, (a), directly, that is, without the addition of any chemical reagents; (b), after it had been subjected to the action of certain chemical reagents, viz: glacial acetic acid, with and without dilution; caustic potash in solution, both weak and strong; chloroform and ether.

2d. This blood was carefully dried upon sterilized cover-glasses by passing these through the flame of a spirit lamp, and then subjected to the action of various staining reagents.

3d. Dengue blood obtained from living subjects was introduced, upon the point of a platinum wire, into test-tubes containing sterilized culture of jelly prepared for this purpose.

These tubes were closed with plugs of sterilized cotton, then placed in an incubator, where the temperature was kept at 100° F. for the growth of such organisms as were contained in the blood.

4th. Blood was drawn directly from the veins of a living subject into a series of sterilized glass bulbs, which were united by a capillary tube. This was performed in such a manner that it seems impossible for germs from the air, or by other accidental means, to have gained an entrance into these bulbs. These were also kept in the incubator, at a temperature of 100° F.

6th. The matters vomited and urine passed by dengue subjects were subjected to microscopic examinations."

Then the Doctor continues on another page:

"When it is remembered that air and water contain large quantities of micro-organisms, that these adhere to the glass apparatus, metal instruments, fingers, and clothing of the operator, and that bacteria wtll quickly form in distilled water, and in staining fluids required in such examinations, the necessity of thoroughly washing and then sterilizing by heat and by chemicals, when this is practicable, all articles used, and by boiling and filtration, or other efficient means, becomes at once apparent.

The methods which I adopted to secure cleanliness and sterilization of all glass apparatus, instruments and other agents used in these experiments, may not have been absolutely perfect in every instance. I made them as uearly so as the means at my command would allow. I am constrained to believe, from the uniformity of the results obtained, notwithstanding some minor defects in the details of my technical methods, that, in the main, they were sufficiently exact to exclude serious errors.

The second difficulty which confronts him who examines blood for schizomycetes, is to dry the blood on the cover-glass without overheating, and thus disintegrating it.

The third difficulty is, to not mistake for micro-organisms certain elements and granules found in normal and pathological blood. These mistakes have occurred frequently, and wrecked many a beautiful hypothesis. That the reader may know and appreciate the difficulties to which I refer, I quote from the recent work of

Dr. F. Huppe: * "The examination of blood for bacteria offers very great difficulty, because in the normal blood within the vessels, and in the normal disintegration of the healthy blood."

Here the Doctor refers in detail to the elements of the blood, which, directly or through their granules, may be confounded with nerve organisms, and continues on page 9 :

"If it be desired to examine the blood for bacteria, a small drop is rapidly spread out in a thin layer, dried, fixed, and then passed three times through the flame, and then stained in the ordinary manner. In such preparations the bacteria are sufficiently stained, but not the granules."

All the glass apparatus and instruments used in the following examinations of dengue blood were sterilized by the following methods: The cover-glass and microscope slides, which had never been previously used, were first washed in warm water with soap, then in clear water, then immersed in Seiler's cleansing fluid, prepared as follows:

| | |
|---|---|
| Bichromate potash ................................ | 2 ozs. |
| Sulphuric acid ........................................ | 3 fld ozs. |
| Water.................................................... | 25 fld ozs. |

When they were taken from this solution, they were again washed in clear water, and finally heated in the flame of a spirit lamp just before they were used. The metal instruments were washed in warm water and soap, clear water, and then in a 5 per cent. solution of carbolic acid; they were also heated in the flame of the lamp just before they were used. The platinum wire was fused to the end of a glass rod ; this wire was sterilized always before using, by heating it to whiteness in the lamp. The arm or finger of the person from whom the blood was obtained was washed with warm water and soap ; then in a 5 per cent. solution of carbolic acid.

The part was then punctured with a sterilized needle, and a small portion of the blood which appeared at the point of puncture was transferred by means of the sterilized platinum wire to the sterilized cover-glass ; this was covered by another glass in order to obtain a thin layer of blood upon each of them ; they were then separated, dried, and finally passed three times through the flame of the lamp, in order to thoroughly coagulate the albumen, and fix the blood elements.

Continuing with the above, the Doctor again goes into interesting and new details, showing the peculiar behavior of these organisms against different chemical re-agents, and on page 14 follows with a description of manipulating the sterilized test tubes. He says:

* "Methods of Bacteriological Investigation," by Dr. Ferdinand Huppe. D. Appleton & Co., New York, 1886.

"When it is considered that these culture investigations occupied a period of six months, and that during this time many transfers occurred from test-tubes containing micrococci to those which did not contain them; that each transfer removed by generations the organisms from those contained in preceding tubes; that frequent microscopic examinations of the different series of tubes invariably revealed a pure culture, *e. g.*, without any other forms of bacteria; it would seem that the methods of sterilization employed had been successful in excluding alien germs, and that those found had a common origin from dengue blood.

The life history of these organisms was watched daily, and studied by means of Holman's life-slide. In the old cultures, those which were removed by many generations from the blood, the organisms were smaller, less deeply colored, and less frequently encapsulated, than those seen in the blood, or removed from it by a few generations. When examined in water without being stained, or in other fluid media which possess a low index of refraction, the micrococci are seen in active movement (Brown movement).

In those cultures which are removed by only a few generations from the blood, the cocci are seen surrounded by capsules. These are sometimes faintly colored pinkish, and can be distinguished from the cocci, which possess a deep color.

Two methods of generation were observed : The first by fission; the organisms are seen to divide through their centres; each coccus will thus form two others. Frequently these newly formed organisms remain united together so as to assume the form of a rod or chaplet; this indicates that they belong to the class named streptococci. In other cases they unite to form swarms or zoogloæ. These latter become more compact, the distinctions of the cocci less, the color of the mass deeper, until they finally contract into corpuscular bodies, about the size of red blood cells; these often unite to form filaments, which frequently assume the shape of a network. This I have often seen when the culture was thinned with distilled water and allowed to dry upon a microscopic slide. This arrangement can be seen whether the specimens are stained or not. The swarms and chaplets of the micrococci can be much better seen in the stained preparations. A histological stand, Abbe's illuminating apparatus and 1-12 H. I. objective, manufactured by J. Grunow, of New York, were used in these researches,

The results obtained in the following and last series of investigations of dengue blood are to me very satisfactory and conclusive, inasmuch as the means adopted to exclude alien germs were, as far as I can see, absolutely perfect.

The apparatus used consisted of a series of glass bulbs united and blown upon a capillary glass tube—Liebig's potash bulbs. To one end of this apparatus was attached a new hypodermic needle, by means of a short piece of new rubber tubing. To the other from this culture bulb, were subjected to the various aniline stains.

The remaining bulbs were examined six weeks later. They all contained pure cultures of the dengue streptococcus, with disintegrated blood cells. These organisms were seen in zoogloœa, swarms, and in compact corpuscular masses; these were often united to form filaments. Normally shaped blood cells were found in only one of these last series of bulbs. I think their preservation in this bulb was due to a layer of coagulated serum which had formed over the surface of its contained blood. Many of the blood corpuscles in this bulb were more or less disintegrated, those which retained their shape were clouded by coagulation of the cell protoplasm, which concealed the organisms they contained to a greater or less extent. These, however, could be seen in great numbers, when the blood was examined in a weak alkaline or acid solution, which rendered the cells more transparent. None of the bulbs, when opened, had the least odor of putrefaction, and the only micro-organisms which they contained were those peculiar to dengue.

With four more pages on this new and interesting subject, Dr. Mc. L. closes his investigations, which, I dare say, are executed with as much care and dexterity as any similar work has been done anywhere outside of Texas, and all those who take the least interest in scientific medicine may justly feel proud over the tedious work and the good results obtained by a Texas physician !

